# Severe Aortic Regurgitation and Ascending Aneurysm in a Patient with Pentacuspid Aortic Valve: Case Report and Review

**DOI:** 10.3390/jcdd12090330

**Published:** 2025-08-28

**Authors:** Nemanja Karamarković, Miloš Grujić, Milica Karadžić, Dejan Lazović, Ivana Đurošev, Mladen J. Kočica

**Affiliations:** 1 Clinic for Cardiac Surgery, University Clinical Center of Serbia, 8th Kosta Todorović St., 11000 Belgrade, Serbia; nemanja_bg89@hotmail.com (N.K.); dr.milosgrujic@gmail.com (M.G.); milica.karadzic@gmail.com (M.K.); lazovic.dejan88@gmail.com (D.L.); ivana.djurosev@gmail.com (I.Đ.); 2 Faculty of Medicine, University of Belgrade, 11000 Belgrade, Serbia

**Keywords:** pentacuspid aortic valve, aortic regurgitation, ascending aortic aneurysm, Bentall procedure, congenital aortic valve anomaly, case report

## Abstract

Pentacuspid aortic valve is an exceptionally rare congenital anomaly that is often associated with functional deterioration and aortopathy. We report a case of a 39-year-old male presenting with severe aortic regurgitation and an ascending aortic aneurysm in the setting of a pentacuspid aortic valve. The patient underwent a successful Bentall and hemiarch replacement using a composite mechanical valved conduit. This case emphasizes the potential association between rare aortic valve morphologies and ascending aortic pathology and includes a brief review of the existing literature on the pentacuspid aortic valve.

## 1. Introduction

Congenital anomalies of the aortic valve (CAAVs) are rare, affecting approximately 1–2% of the general population [1,2,3,4]. Pentacuspid aortic valve (PAV), defined by the presence of five aortic cusps, is the rarest form, with an estimated prevalence of less than one in a million people [2]. Most of the very few reported PAV cases (Supplementary Materials: Table S1) are associated with valvular dysfunction, primarily aortic regurgitation (AR), and a subset are complicated by ascending aortic aneurysms [5,6,7,8,9,10,11,12,13,14,15,16,17]. The pathophysiological mechanisms linking abnormal cusp morphology and aortopathy remain incompletely understood, but they may share features with those seen in bicuspid aortic valve (BAV)-related aortopathy [3,18,19].

This case report describes a patient with severe AR and an ascending aortic aneurysm caused by a functionally pentacuspid valve and discusses its clinical and surgical implications in the context of the available literature.

## 2. Case Presentation

A 39-year-old male presented with symptoms of exertional dyspnea, orthopnea, and episodes of shortness of breath. Ten months prior to admission, he experienced pulmonary edema, which was managed medically at a regional hospital. The patient had no known comorbidities but was an active smoker.

Upon admission, he was normotensive (blood pressure 120/70 mmHg) and receiving the following medications: bisoprolol 2.5 mg once daily, ramipril 2.5 mg once daily, atorvastatin 20 mg once daily, furosemide once daily, and spironolactone 25 mg once daily. The electrocardiogram showed sinus rhythm with a heart rate of 76 beats per minute.

**Figure 1:**
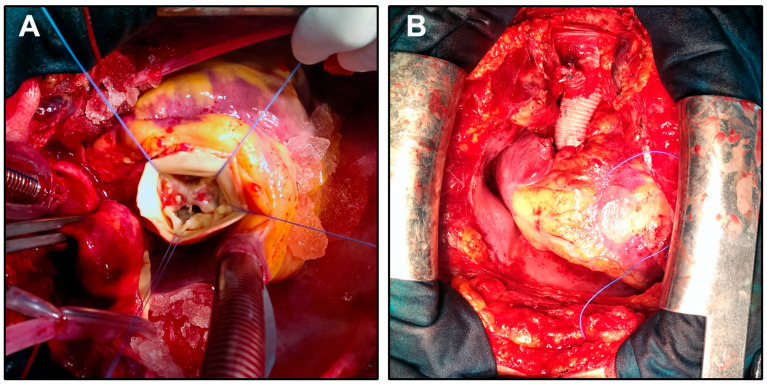
Intraoperative views during surgical correction of a PAV and ascending aortic aneurysm. (**A**) Intraoperative exposure of a PAV during aortic root replacement. The valve shows five distinct cusps with asymmetric morphology. Stay sutures are placed to retract the aortic wall and expose the valve anatomy. (**B**) Completion of the procedure following valve and ascending aorta replacement with a composite graft. The graft anastomosis and surrounding structures are shown in situ prior to chest closure.

**Figure 2:**
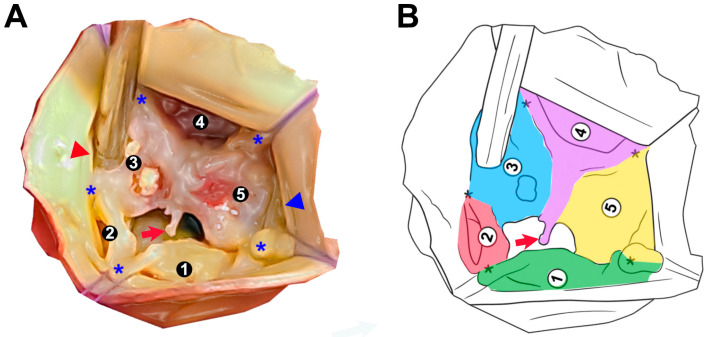
Morphological anatomy of a pentacuspid aortic valve (PAV): surgical and schematic views. (**A**) Intraoperative photograph demonstrating a severely dysmorphic PAV with five identifiable cusp regions labeled 1–5 (the same as Figure 1A but zoomed in and cropped). Note the irregular cusp sizes, variable commissural spacing, and presence of fused and rudimentary cusp tissue. Blue asterisks (*) denote commissures and pseudocommissures. The red arrow indicates the presence of an anomalous cord extending from the raphe of the conjoined cusp. The red triangle depicts the position of the LMCA ostium. The blue triangle indicates the position of the RCA ostium. (**B**) Corresponding schematic illustration of the valve morphology shown in (**A**), highlighting the asymmetry and heterogeneous cusp arrangement. Each cusp is distinctly color-coded (green, red, blue, purple, and yellow) and labeled for correlation with panel (**A**). Note the fusion between cusps 3, 4, and 5, as well as the underdeveloped morphology of cusp 2.

Transthoracic echocardiography (TTE) revealed a left ventricular end-diastolic diameter (LVEDD) of 65 mm and an end-systolic diameter (LVESD) of 48 mm, with a preserved ejection fraction (LVEF) of 56%. The aortic valve was severely calcified, with cusp fusion and decreased mobility (Figure 1A and Figure 2A). Severe aortic regurgitation (grade 4+) was present, with an effective regurgitant orifice area (EROA) of 0.41 cm^2^, a regurgitant volume (RV) of 65 mL/beat, and a regurgitant fraction (RF) of 52% [20]. The aortic annulus measured 27 mm, the sinotubular junction 40 mm, and the ascending aorta 54 mm. Suprasternal and high right parasternal views demonstrated normal diameters of the aortic arch and descending thoracic aorta, with no evidence of dilation, dissection, or coarctation. Mild mitral and tricuspid regurgitations (1+) were observed, with a systolic pulmonary artery pressure of 58 mmHg. The aortic valve was functionally described as bicuspid, though leaflet morphology was not clearly defined. Coronary angiography revealed a normal branching pattern of the coronary arteries and the absence of obstructive lesions.

The patient underwent surgery via anterograde partial cardiopulmonary bypass (CPB). A Bentall procedure with hemiarch replacement was performed using a composite On-X Ascending Aortic Prosthesis (25 mm) with Vascutek Gelweave Valsalva Graft (26 mm) (Terumo Cardiovascular Systems, Ann Arbor, MI). The cardiopulmonary bypass time was 185 min, the aortic cross-clamp (ACC) time was 105 min, and the circulatory arrest (CA) time was 10 min (open distal anastomosis) at a core temperature of 19 °C and jugular venous oxygen saturation of 100% (Figure 1). Intraoperative transesophageal echocardiography (TEE) showed normal prosthetic valve function and satisfactory hemodynamic performance.

Intraoperative aortic root analysis (Figure 2) revealed a severely dysmorphic PAV with five identifiable cusp regions of irregular sizes and partial fusion, predisposing central malcoaptation and eccentric aortic regurgitation. Cusp 1 (green) appears well-developed and free-standing, corresponding with the non-coronary cusp of the normal tricuspid valve, with true commissures towards neighboring cusps. Cusp 2 (red) is the smallest accessory cusp. Between cusps 3 (blue), 4 (purple), and 5 (yellow), there appears to be a fusion zone forming raphe-like structures. The commissures between these cusps are asymmetric and seem shallower or less developed, which is consistent with pseudocommissures [4]. Partial cusp fusion is visible between cusps 2 and 3. An anomalous cord, extending from the raphe of the conjoined cusp, protrudes into an eccentric orifice [21]. The present morphology with cusp asymmetry and fusion is likely to exhibit bicuspid physiology, as seen on TEE.

Both coronary ostia were of normal size and free of atherosclerotic disease. In relation to the asymmetric PAV anatomy, the left main coronary artery (LMCA) ostium had a slightly lower take-off and corresponded to cusp 3. The right coronary artery (RCA) ostium had a normal take-off, corresponding to cusp 5, and was positioned slightly closer to the adjacent commissure (between cusp 1 and cusp 5) than in a typical tricuspid configuration. Coronary flow was unaffected, as demonstrated by preoperative angiography and confirmed intraoperatively by unimpeded cardioplegia delivery (Figure 2A).

The postoperative period was marked by prolonged leukocytosis and neutrophilia (until postoperative day 6), with leukocyte counts up to 19 × 10^9^/L and neutrophil counts up to 85%. A de-escalation antibiotic regimen including vancomycin and meropenem was administered. The patient remained afebrile throughout hospitalization. Urine and blood cultures, as well as surgical wound swabs, were negative for infection. Upon normalization of white blood cell counts (Leu 9.1 × 10^9^/L; Neu 65.7%), the patient was discharged on postoperative day 13 in a stable hemodynamic condition and without signs of systemic or local infection.

At the 11-year follow-up, the patient remains in good general condition with normal function of the aortic prosthesis and preserved left ventricular systolic function.

## 3. Discussion

Structural CAAVs affect approximately 1–2% of the general population, with BAV being the most common, occurring in 0.5–1.4% of individuals [22,23]. Unicuspid aortic valve (UAV) is considerably rarer, with an estimated prevalence of 0.02% [24], while a quadricuspid aortic valve (QAV) is observed in 0.003–0.013% of the population [25]. In contrast, PAV is extraordinarily rare, with only isolated case reports in the literature and no robust epidemiological data, suggesting a prevalence likely well below 0.0001%, or fewer than 1 in 1 million [4]. This extreme rarity has precluded formal population-based prevalence estimates and highlights the need for systematic reporting and surveillance.

A review of 14 reported PAV cases (Supplementary Materials: Table S1) reveals a consistent association between the young age (24–39 years), asymmetric cusp morphology, severe eccentric AR, and aortic dilatation, particularly of the root or ascending aorta, in four (28.6%) cases, including the current report [6,9,10]. Of those, three cases underwent root and ascending aorta reconstructions (i.e., Robicsek and Bentall), in addition to AV replacement [9,10]. In our case, the patient underwent a Bentall procedure with hemiarch replacement, which remains the treatment of choice in patients with combined valvular insufficiency and ascending aortic aneurysm [26]. In this case, the decision to use a mechanical composite graft was appropriate given the patient’s young age, preserved ventricular function, and the need for durable correction. The uneventful recovery and excellent 11-year follow-up outcome reinforce the long-term efficacy of this approach when tailored appropriately.

The observed asymmetry, including a rudimentary cusp (cusp 2) and raphe formation between cusps 3, 4, and 5 (Figure 2), supports the hypothesis that cusp malformation and fusion contribute to poor coaptation and progressive AR [4]. In addition, the anomalous cord arising from the raphe’s free margin and floating within the regurgitant orifice (Figure 2) adds a unique structural abnormality not previously described with PAV. A similar structure, with its free portion attached to the aorta, has been documented in some BAV cases, contributing to chronic and acute AR [21].

The co-occurrence of root and ascending aortic dilatation in current and previously reported cases contributes to the hemodynamic burden on the aortic wall, either directly (via eccentric regurgitant jets) or indirectly (through altered flow patterns and wall stress), promoting aneurysmal formation and remodeling [4]. There is also accumulating evidence that CAAVs share some genetics and embryological defects involving both valvulogenesis and aortic wall maturation, predisposing individuals to aortopathy. A rare histological examination of the resected PAV demonstrated myxomatous degeneration, which may support a possible underlying connective tissue abnormality [27].

Genetic studies in BAV and associated aortopathy have implicated a limited number of genes, most notably NOTCH1, SMAD6, and ACTA2. In a large BAV/TAA cohort, SMAD6 variants were specifically enriched among patients with concurrent thoracic aortic aneurysms [28,29], while NOTCH1 and ACTA2 mutations were also documented in familial or heritable forms of aortic valve disease and aortic wall pathology [30]. However, no analogous genetic associations have been reported for PAV, which remains exceedingly rare and lacks familial or syndromic clustering. Nevertheless, in patients with PAV and concomitant ascending aortic dilation, it is plausible that similar developmental pathways affecting valve morphogenesis and aortic wall structure may be involved, and an echocardiographic screening of first-degree relatives may be reasonable in selected cases.

The decision to perform a Bentall procedure in this patient was multifactorial. First, although preoperative sinus of Valsalva measurements were unavailable, we did not expect a normal root configuration, and this was confirmed by intraoperative assessment. Second, while no definitive association between PAV and aortopathy has been demonstrated, the possibility of intrinsic root wall pathology and future dilation could not be excluded. Third, technical challenges were anticipated in adapting commercially available tubular grafts to a markedly dilated STJ proximally and a normal-sized arch distally. Finally, the use of a composite Valsalva graft offered the advantage of addressing all pathologies in a single reconstruction while restoring near-physiological root geometry, a procedure well within the extensive experience of our surgical team.

Associated congenital anomalies were reported in two cases: patent foramen ovale [8] and renal artery dysplasia [13].

Despite the rarity of PAV, this case underscores the importance of considering it in the differential diagnosis of unexplained AR, especially in young people and when associated with ascending aortic dilation. Preoperative recognition remains difficult but may improve with advances in imaging techniques such as 3D echocardiography, MDCT, and cardiac MRI, which can better delineate the commissural architecture and leaflet morphology [31].

## 4. Conclusions

Pentacuspid aortic valve represents an exceptionally rare congenital anomaly, most often presenting with asymmetric cusp morphology, severe aortic regurgitation, and, in a subset of patients, ascending aortic dilatation. A review of the literature reveals a reproducible pattern of functional insufficiency and structural aortopathy, frequently necessitating combined valvular and aortic surgical intervention. These findings suggest a pathophysiological overlap with BAV-associated aortopathy and support the notion that PAV should be approached as a valvulo-aortic disease entity.

However, current understanding is severely limited by the rarity of PAV, lack of centralized registries, heterogeneous reporting, and scarce histological or genetic characterization. As a result, long-term outcomes, optimal timing of intervention, and risk stratification remain largely undefined.

Future efforts should prioritize the development of multicenter registries and standardized morphological classification schemes. Advanced imaging techniques may enhance preoperative recognition, while systematic histological and molecular investigations could uncover developmental or genetic substrates underlying PAV and its associated aortopathy. Such coordinated strategies are essential to changing PAV from an anecdotal curiosity to a better-understood clinical and surgical entity.

## Data Availability

Data are contained within the article.

## Supplementary Materials

The following supporting information can be downloaded at https://www.mdpi.com/article/10.3390/jcdd12090330/s1, Table S1: A literature review of the reported PAV cases.

## Abbreviations

The following abbreviations are used in this manuscript:

CAAVs	Congenital Anomalies of the Aortic Valve
PAV	Pentacuspid Aortic Valve
AR	Aortic Regurgitation
BAV	Bicuspid Aortic Valve
TEE	Transthoracic Echocardiography
LVEDD	Left Ventricular End-Diastolic Diameter
LVESD	Left Ventricular End-Systolic Diameter
LVEF	Left Ventricular Ejection Fraction
EROA	Effective Regurgitant Orifice Area
RV	Regurgitant Volume
RF	Regurgitant Fraction
CPB	Cardiopulmonary Bypass
ACC	Aortic Cross-Clamp
CA	Circulatory Arrest
TEE	Transesophageal Echocardiography
LMCA	Left Main Coronary Artery
