# Supplementary material for: Severe Aortic Regurgitation and Ascending Aneurysm in a Patient with Pentacuspid Aortic Valve: Case Report and Review
Source: J Cardiovasc Dev Dis. 2025 Aug 28;12(9):330. doi: 10.3390/jcdd12090330 (PMC12471185; doi:10.3390/jcdd12090330)
Supplement: Supplementary file 1 [file jcdd-12-00330-s001.zip › Table S1 - Literature review of the reported PAV cases..pdf]

Table S1 – Literature review of the reported PAV cases.

Year	Author	No	Age	Gender	Morphology	Function	Diagnosis	Treatment	Other congenital	Aorta	Histology
1923	**Simonds** [5]	1	NA	NA	NA	NA	NA	NA	NA	NA	NA
1982	**Bogers** [6]	1	24	M	Asymmetric	AS, AR (moderate)	CXR, Cath.	AVR	None	Dilated (root)	NA
1984	**Yates** [7]	1	62	F	NA	Severe AR	NA	AVR	NA	NA	NA
2000	**Cemri** [8]	1	19	M	Asymmetric	Normal function	TTE, TEE	None	PFO	NA	NA
2009	**Meng** [9]	1	24	M	Symmetric	Severe AR	TTE, MDCT, MRI	AVR, Robicsek	None	Dilated (asc.)	NA
2010	**Wang [**10]	1	24	M	Asymmetric	Severe AR	TTE, MDCT	AVR, Robicsek	None	Dilated (root, asc.)	NA
2012	**Kuroki** [11]	1	46	M	Asymmetric	Severe AR	TTE, TEE	AVR	NA	NA	Myxomatous
2015	**Ozyilmaz** [12]	1	48	F	NA	Severe AR	3D TEE	AVR	NA	NA	NA
2020	**Patanè** [13]	1	26	F (p)	Asymmetric	Severe AR	TTE, TEE	AVR	Renal aa. dysplasia	NA	Myxomatous
2021	**Ansari** [14]	1	54	M	Asymmetric	Mid-severe AR	TTE, TEE	AVR	NA	Normal	NA
2022	**Beddingfield** [15]	1	56	F	Asymmetric	Severe AR	TEE	AVR	NA	NA	NA
2022	**Motoki** [16]	1	52	F	Asymmetric	Severe AR	TTE, TEE	AV Repair	NA	NA	NA
2023	**Albogmi** [17]	1	54	M	Asymmetric	Mid-severe AR	TEE	AVR	None	NA	NA
2025	**Karamarkovic** *	1	39	M	Asymmetric	Severe AR	TTE, MDCT, TEE	Bentall and hemiarch	None	Dilated (root, asc.)	NA

* Current Report; F(p) – pregnant female; AS – Aortic Stenosis; AR – Aortic regurgitation; TTE – Transthoracic echocardiography; TEE – Transesophageal echocardiography; MDCT – Multi Detector Computerized Tomography; CXR – Chest X-Ray; Cath. – Catheterization; PFO – Patent Foramen Ovale.

References: Reference numbers in the table correspond to reference numbers in the main text.
